# Left bundle branch area pacing: How to prevent a coronary venous fistula

**DOI:** 10.1002/joa3.12845

**Published:** 2023-03-26

**Authors:** Mattia Strazzanti, Giacomo Mugnai, Leonardo Marinaccio, Paolo Alberto Del Sole, Luca Tomasi

**Affiliations:** ^1^ Division of Cardiology, Department of Cardiovascular and Thoracic Sciences, School of Medicine University of Verona Verona Italy; ^2^ Department of Cardiology Immacolata Concezione Hospital Piove di Sacco Padova Italy

**Keywords:** left bundle branch pacing area, septal veins

In patients with decreased left ventricular ejection fraction (LVEF) and the need of cardiac pacing for an advanced AV block, the left bundle branch area pacing (LBBAP) has emerged as an effective tool to promote the physiological pacing of conduction system in order to prevent the detrimental effects of a permanent right ventricular pacing. Although coronary vein fistulas seem to be rare and harmless, literature is sparse about this complication. The first two cases describe the occurrence of coronary vein fistulas; in the last case, we show a simple method which can be helpful to avoid the breach of septal veins during LBBAP implantation.

Following a successful transcatheter aortic valve implantation (TAVI), an 83‐year‐old male experienced a complete AV block. As the patient had a moderate reduction of LVEF, he was implanted with a LBBAP. After positioning the lead via the designed 3D pacing‐tool system which included a specific delivery sheath (Selectra 3D, Biotronik) and an extandable‐retractable active screw, stylet‐driven pacing lead (Solia S60, Biotronik), left bundle capture was achieved and proven by the expected QR morphology in lead V_1_ (pacing at higher output), narrowing of the QRS and reasonable left ventricular activation time. As our common clinical practice for LBBAP implantation, the contrast injection was used to assess the lead depth through the interventricular septum. The angiography—performed in right anterior oblique projection (Figure 1A)—showed that the contrast dye filled a septal vein which went upward and posteriorly and then joined the coronary sinus. No symptoms, no EKG changes, and no pericardial effusion were detected immediately and after the procedure. As the parameters of amplitude and threshold were optimal, the lead was not retracted. All the electric parameters remained stable at the end of the procedure and the day after. The patient never experienced chest pain and the troponin test showed no myocardial impairment.

**FIGURE 1:**
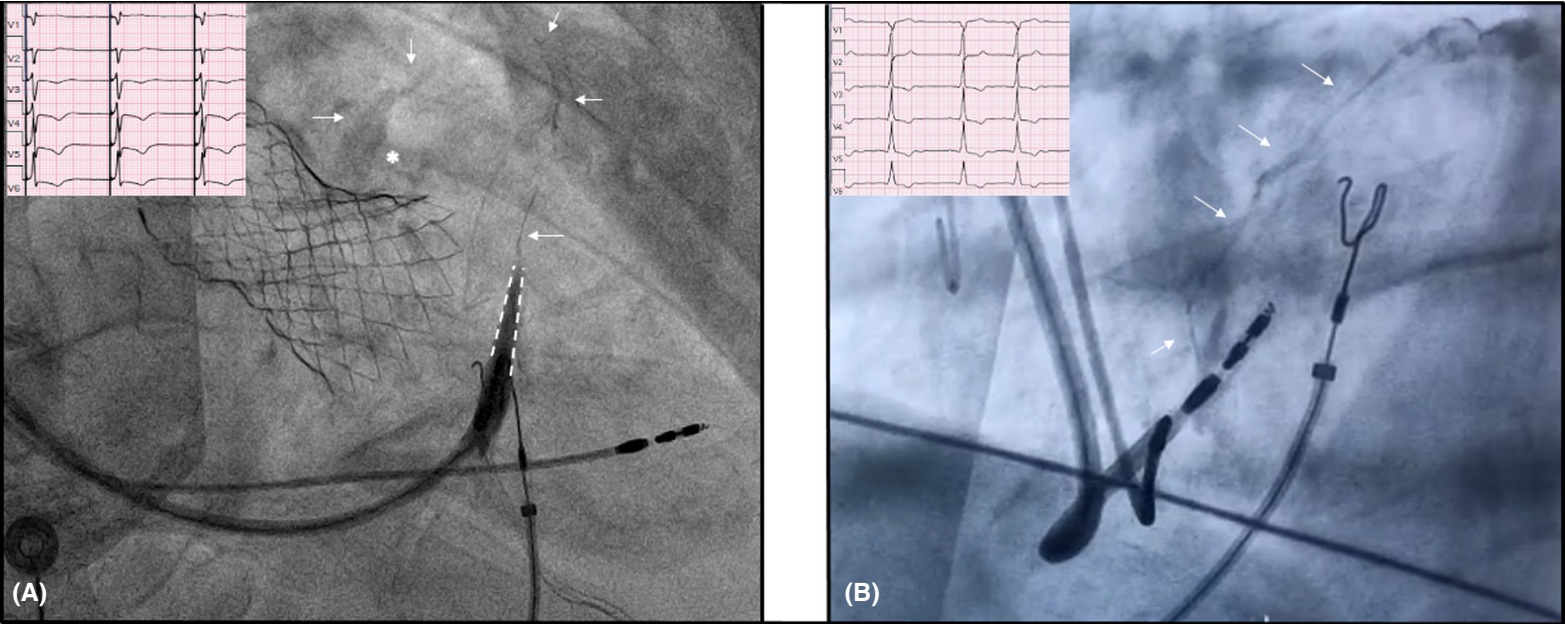
(A) Right anterior oblique projection 30° showing the left bundle branch pacing catheter through the septum (dashed white lines) and the contrast filling the coronary vein fistula (white arrows) reaching the coronary sinus (white asterisk). In a small box, the ECG precordial leads show LBBAP. (B) Left anterior oblique projection at 22° showing the left bundle branch pacing catheter screwed through the septum, the contrast filling the perforator branch vein (white arrows) until to the great cardiac vein of the coronary sinus. In a small box, the ECG precordial leads show LBBAP.

The second case deals with an 84‐year‐old patient who was admitted for advanced AV block associated with moderate depression of the LVEF due to ischemic heart disease. Based on the basal QRS of 130 ms and the presumptive high percentage of pacing in a left ventricular dysfunction, the LBBAP was attempted. The lead was placed using the abovementioned 3D pacing‐tool system and a capture of the left bundle branch area was achieved (Figure 1B). From the right ventricle, the lead was inserted through the septum with 7–8 clockwise turns. After penetration of the lead, contrast was injected through the sheath, filling the perforator branch vein and reaching the great cardiac vein of the coronary sinus (Figure 1B, in left anterior oblique projection). The electronic parameters were fine (sensed R‐wave 11.5 mV, capture threshold 0.6 V@0.4 ms, impedance 730 Ω), no symptoms and no EKG changes occurred, no signs of pericardial effusion were detected. As the parameters of amplitude and threshold were optimal, the lead was not retracted. All the electric parameters remained stable at the end of the procedure and the day after. The following hospital stay was unremarkable.

Finally, a 76‐year‐old patient was referred for pace and ablate strategy because of atrial fibrillation with high ventricular rates and mildly depressed left ventricular ejection fraction. Before placing the lead for LBBAP, positioning the whole system (Selectra 3D, Biotronik) across the tricuspid valve and gently leaning on the septum, contrast injection was applied in order to map and detect possible coronary septal vein in order to avoid it during lead implantation (Figure 2A and Video S1). In this case, the contrast injected through the sheath was able to visualize a septal vein which joined to coronary sinus (Figure 2A and Video S1). Later on, the lead was implanted in the LBBAP avoiding the region of septal branch which was previously identified (Figure 2B).

**FIGURE 2:**
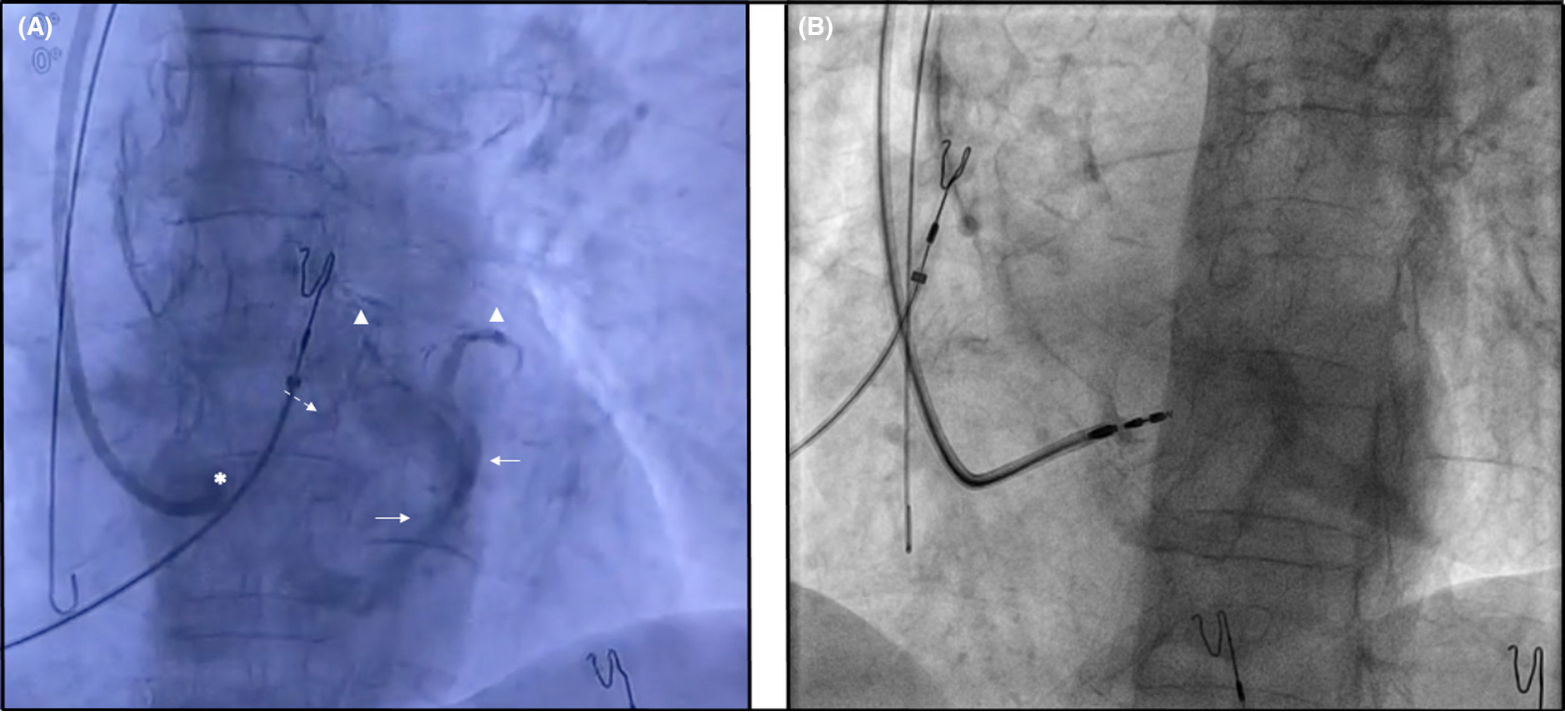
(A) Anteroposterior projection showing contrast injection through the sheath which was positioned across the tricuspid valve and gently leaned to the septum (white asterisk). The contrast injection showed the route of a small septal vein (dashed arrow) joining to the coronary sinus (white arrows) and highlighting the anterior and antero‐lateral branches of coronary sinus (white triangles). (B) Left anterior oblique projection showing the final screw of the lead through the septum obtaining LBBAP without breaching any septal veins.

Breaching a septal vein during the catheter positioning is a rare—but probably underdiagnosed—complication of the LBBAP implantation, unlikely to be harmful according to the few cases described.^1^, ^2^, ^3^ A recent large registry‐based observational multicenter study reported 7 cases of coronary vein fistulas out of 2533 LBBAP procedures (0.28%).^1^ In addition, two case reports had previously described this complication.^2^, ^3^ In all these reported cases, no serious consequences followed. In all the cases, given the small size and asymptomatic nature of the fistula, no treatments were needed and a conservative follow‐up was enough. Of note, extendable‐retractable active screw stylet‐driven pacing leads were used in the present cases. Although no evidence is still reported in literature, smaller diameter lumen‐less leads might hypothetically reduce the risk of this kind of complications.

This complication might potentially be underestimated as not all centers use contrast during LBBAP. Septal coronary vein infringements probably result from the transeptal route of the LBBAP lead; the contrast injected through the sheath might show possible ways from small septal branches to the coronary sinus. The coronary vein fistula was confirmed by the persistent blood flow from the right ventricle to the coronary sinus still shown at the end of both procedures. In the last case, we describe a simple method in order to avoid to breach small septal veins of coronary sinus. Positioning the long sheath across the tricuspid valve and gently leaning on the septum, the contrast injection is able to visualize the route of small perforator veins inside the septum. This information can be very precious during implantation of LBBBAP in order to avoid to screw the lead through a septal veins leading to a coronary venous fistula.

Although all the reports of coronary vein fistulas describe a benign course without serious harms for the patient,^1^, ^2^, ^3^ the last case emphasizes a possible role of the contrast agents before LBBAP, in order to assess possible routes of small perforator veins through the septum helping to avoid them during lead implantation.

## CONFLICT OF INTEREST STATEMENT

The authors declare no conflicts of interest.

## ETHICS APPROVAL STATEMENT

N/A.

## PATIENT CONSENT STATEMENT

Signed.

## CLINICAL TRIAL REGISTRATION

N/A.

## Supporting information


Figure S1.

Video S1.
