# Supplementary figures and images for: Left bundle branch area pacing: How to prevent a coronary venous fistula
Source: J Arrhythm. 2023 Mar 26;39(3):491–3. doi: 10.1002/joa3.12845 (PMC10264724; doi:10.1002/joa3.12845)

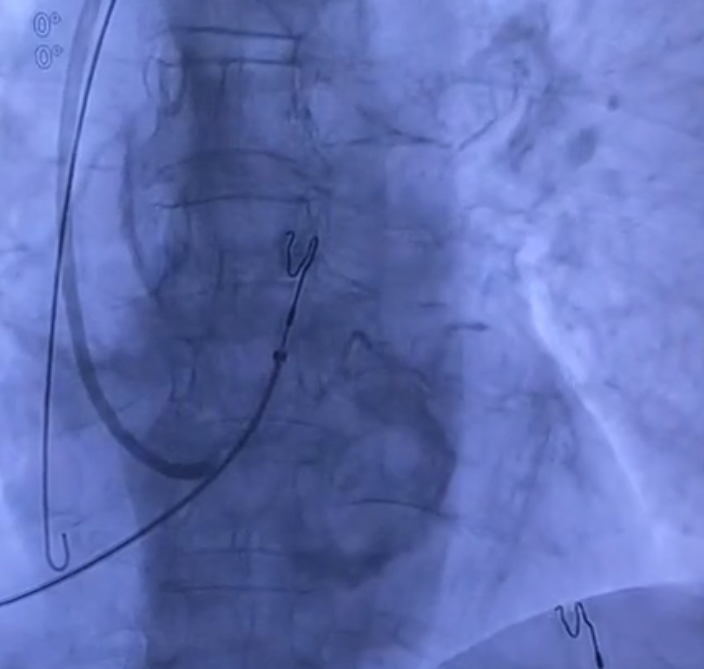

Supplement: Supplementary file 1 — Figure S1. [file JOA3-39-491-s001.jpg]
